# Supplementary material for: New azobenzene liquid crystal with dihydropyrazole heterocycle and photoisomerization studies
Source: R Soc Open Sci. 2020 Jul 1;7(7):200474. doi: 10.1098/rsos.200474 (PMC7428282; doi:10.1098/rsos.200474)
Supplement: Detailed procedures of synthesis and characterization of compounds 2a - 2c, Fig. S1 - S6 (DSC curves no shown in text), Fig. S7 - S34 (NMR), Fig. S35 - S45 (HRMS). [file rsos200474supp1.pdf]

Electronic Supplementary Information

New azobenzene liquid crystal with dihydropyrazole heterocycle and photoisomerisation studies

Xiaoxuan Wang, Zhaoxia Li, Haiying Zhao*and Shufeng Chen

Inner Mongolia Key Laboratory of Fine Organic Synthesis, College of Chemistry and Chemical Engineering, Inner Mongolia University, Hohhot 010021, China.

*Corresponding author. Fax: +86 471 4992982.

E-mail addresses: hyzhao@imu.edu.cn

Measurements

IR spectra were recorded as KBr pellets on a Bruker-ALPHA spectrometer. NMR spectra were recorded on an Avance 500 Bruker (500 MHz) spectrometer using tetramethylsilane as internal standard. HRMS spectra were recorded on a Bruker ultrafleXtreme MALDI-TOF/TOF mass spectrometer. DSC thermographs were obtained on a METTLER TOLEDO DSC3 at a heating rate of 5 ˚C min^{-1} under nitrogen flow.

General procedures of synthesis and characterization of compounds 2a-2c

To a stirred solution of α,β-unsaturated diketone **1** (1.5 mmol) in ethanol (5 mL) was charged with hydrazine hydrate (80%, 1.96 g, 61.3 mmol). The resulting mixture was heated at reflux for 30 min, and then filtered at reduced pressure to yield a orange viscous liquid. The unstable intermediate was immediately dissolved in CH_2Cl_2 (5 mL). To the above solution was added a solution of acetyl chloride (0.184 g, 2.35 mmol) dropwise at 20 ℃. The resulting mixture was further stirred for 10 min. The reaction mixture was washed with water and charged with CH_2Cl_2, then partitioned between H_2O and CH_2Cl_2. The organic extract was dried ($MgSO_4$) and concentrated, which was further purified by silica gel chromatography to yield **2** as a yellow powder.

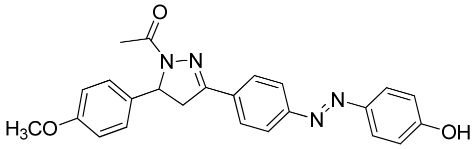

2a, yield 55%. m. p. 237~240℃; ^{1}H NMR (500 MHz, DMSO) δ 10.42 (s, 1H), 7.95 (d, J = 11.0 Hz, 2H), 7.88 (d, J = 10.5 Hz, 2H), 7.84 (d, J = 11.0 Hz, 2H), 7.13 (d, J = 11.0 Hz, 2H), 6.96 (d, J = 11.0 Hz, 2H), 6.88 (d, J = 11.0 Hz, 2H), 5.52 (dd, 1H), 3.87 (dd, 1H), 3.72 (s, 3H), 3.17 (dd, 1H), 2.32 (s, 3H). ^{13}C NMR (125 MHz, $CDCl_3$) δ 167.43, 161.36, 158.42, 153.44, 152.73, 145.34, 134.38, 132.82, 127.68, 126.83, 125.13, 122.52, 116.04, 113.98, 59.18, 55.08, 42.00, 21.81; IR (KBr) v: 3421, 3129, 2925, 1630, 1567, 1509, 1458, 1243, 1133, 1029, 846, 555 cm^{-1}; HRMS m/z: Calcd for $C_{24}H_{23}N_4O_3$ $[M+H]^+$ 415.1770, found 415.1768.

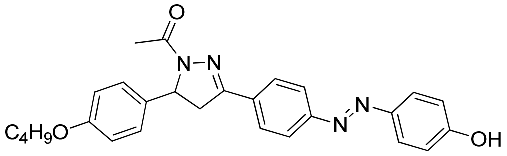

2b, yield 42.7%. m.p. 238~240℃; ^{1}H NMR (500 MHz, DMSO) δ 10.42 (s, 1H), 7.95 (d, J = 11.0 Hz, 2H), 7.88 (d, J = 10.5 Hz, 2H), 7.84 (d, J = 11.0 Hz, 2H), 7.13 (d, J = 11.0 Hz, 2H), 6.96 (d, J = 11.0 Hz, 2H), 6.88(d, J = 11.0 Hz, 2H),

5.52 (dd, 1H), 3.92 (t, J = 8.5 Hz, 2H), 3.86 (dd, 1H), 3.19 (dd, 1H), 2.32 (s, 3H), 1.68 ~1.63 (m, 2H), 1.44 ~ 1.36 (m, 2H), 0.91 (t, J = 9.5Hz, 3H); ^{13}C NMR (125 MHz, DMSO) δ167.88, 161.83, 158.34, 153.91, 153.20, 145.81, 134.70, 133.29, 128.14, 127.27, 125.59, 122.99, 116.51, 114.93, 67.55, 59.63, 42.45, 31.18, 22.27, 19.19, 14.14; IR (KBr) v: 3423, 2917, 2848, 1679, 1608, 1513, 1400, 1265, 1170, 1078, 848, 557 cm^{-1}; HRMS m/z: Calcd for $C_{27}H_{28}N_4O_3Na^+$ 479.2059 [M+Na]$^+$, found 479.2040.

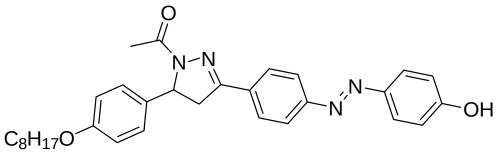

2c, yield 42%. m.p. 209~210 ℃; ^{1}H NMR (500 MHz, DMSO) δ 10.42 (s, 1H), 7.95 (d, J = 11.0 Hz, 2H), 7.88 (d, J = 10.5 Hz, 2H), 7.84 (d, J = 11.0 Hz, 2H), 7.13 (d, J = 11.0 Hz, 2H), 6.96 (d, J = 11.0 Hz, 2H), 6.88 (d, J = 11.0 Hz, 2H), 5.52 (dd, 1H), 3.90 (t, J = 8.0 Hz, 2H), 3.86 (dd, 1H), 3.17 (dd,1H), 2.32 (s, 3H), 1.70 ~ 1.63 (m, 2H), 1.42~1.33 (m, 2H), 1.33 ~ 1.17 (m, 8H), 0.91 (t, J = 9.0 Hz, 3H); ^{13}C NMR (125 MHz, DMSO)) δ 167.41, 161.36, 157.87, 153.43, 152.73, 145.35, 134.22, 132.82, 127.67, 126.80, 125.12, 122.52, 116.04, 114.46, 67.39, 59.17, 41.99, 31.24, 28.73, 28.67, 25.52, 22.08, 21.80, 13.95; IR (KBr) v: 3423, 3056, 2923, 2850, 1629, 1579, 1463, 1240, 1133, 842, 549 cm^{-1}; HRMS m/z: Calcd for $C_{31}H_{36}N_4O_3Na^+$ 535.2680 [M+Na]$^+$, found 535.2635.

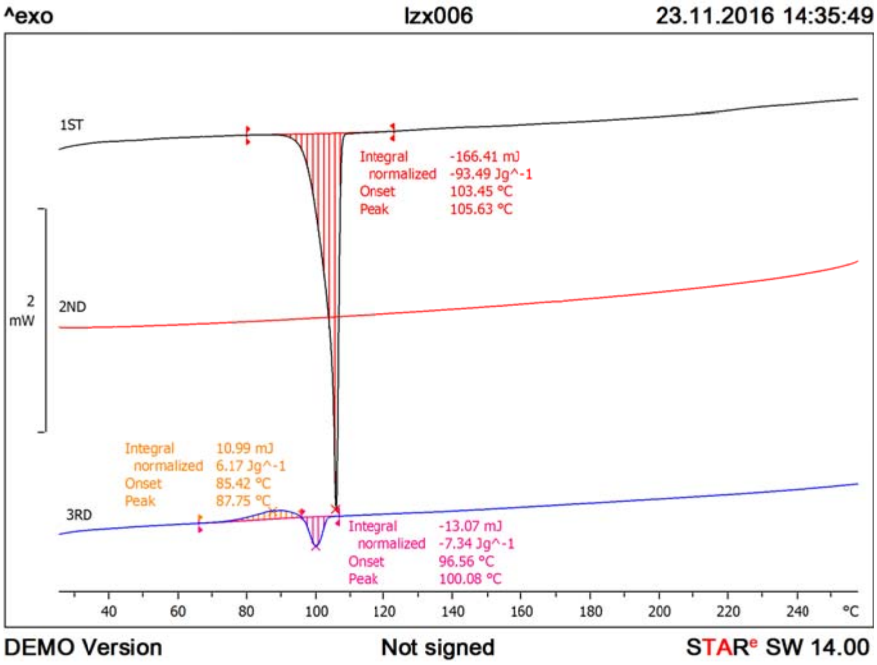

Fig. S1 DSC curve of compound **3a-8**

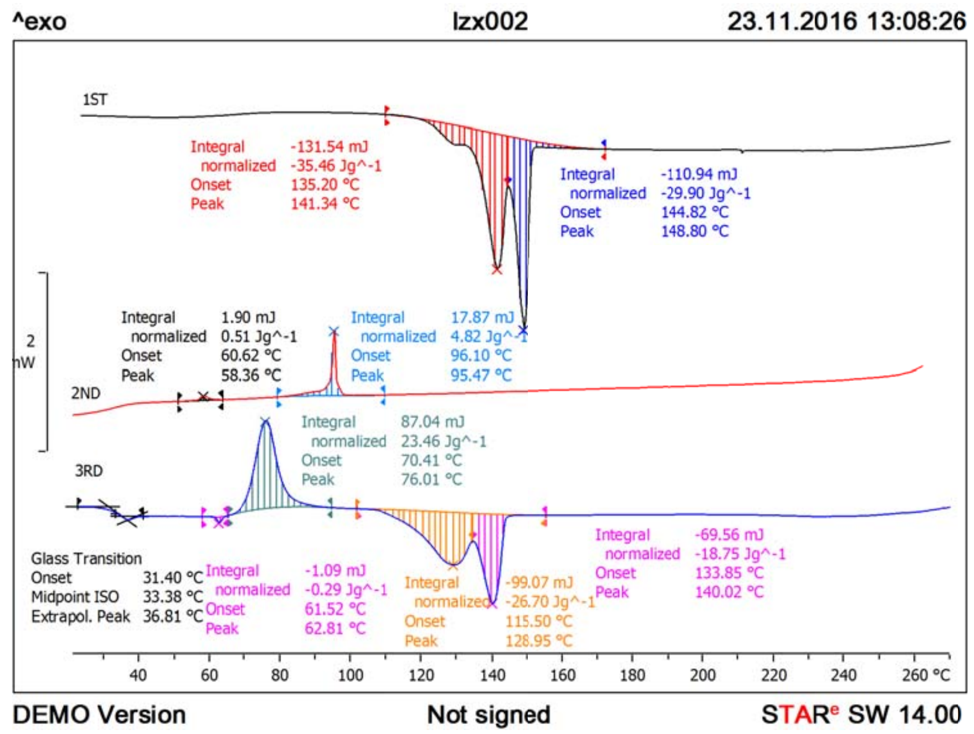

Fig. S2 DSC curve of compound **5a-8**

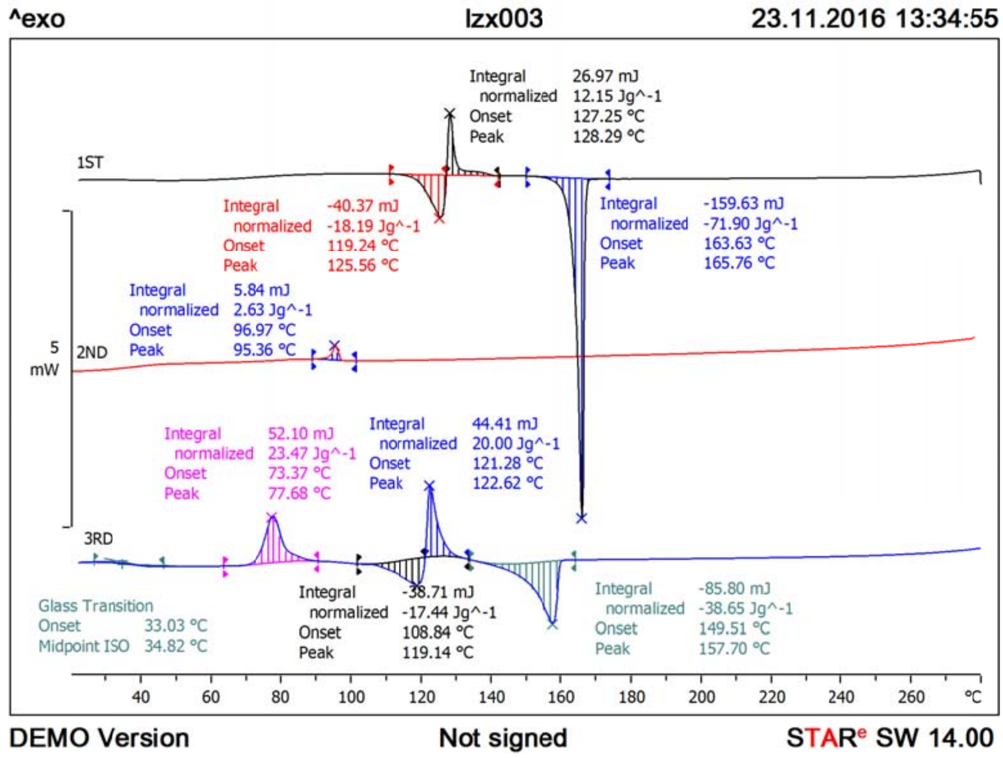

Fig. S3 DSC curve of compound **5a-10**

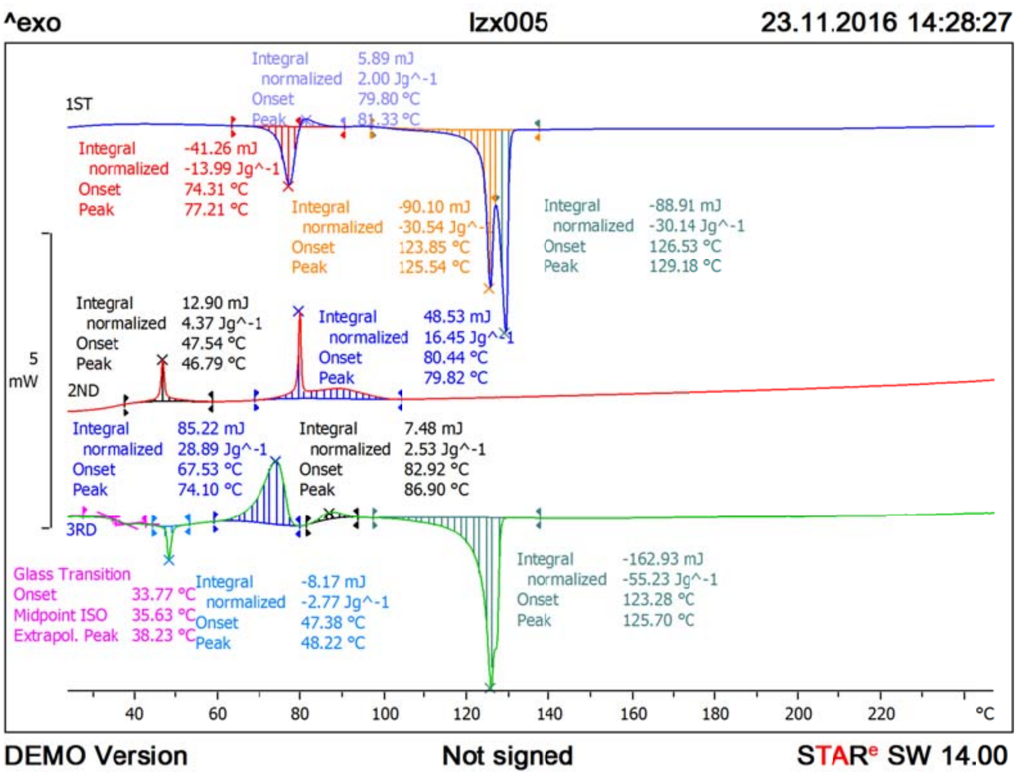

Fig. S4 DSC curve of compound **5a-16**

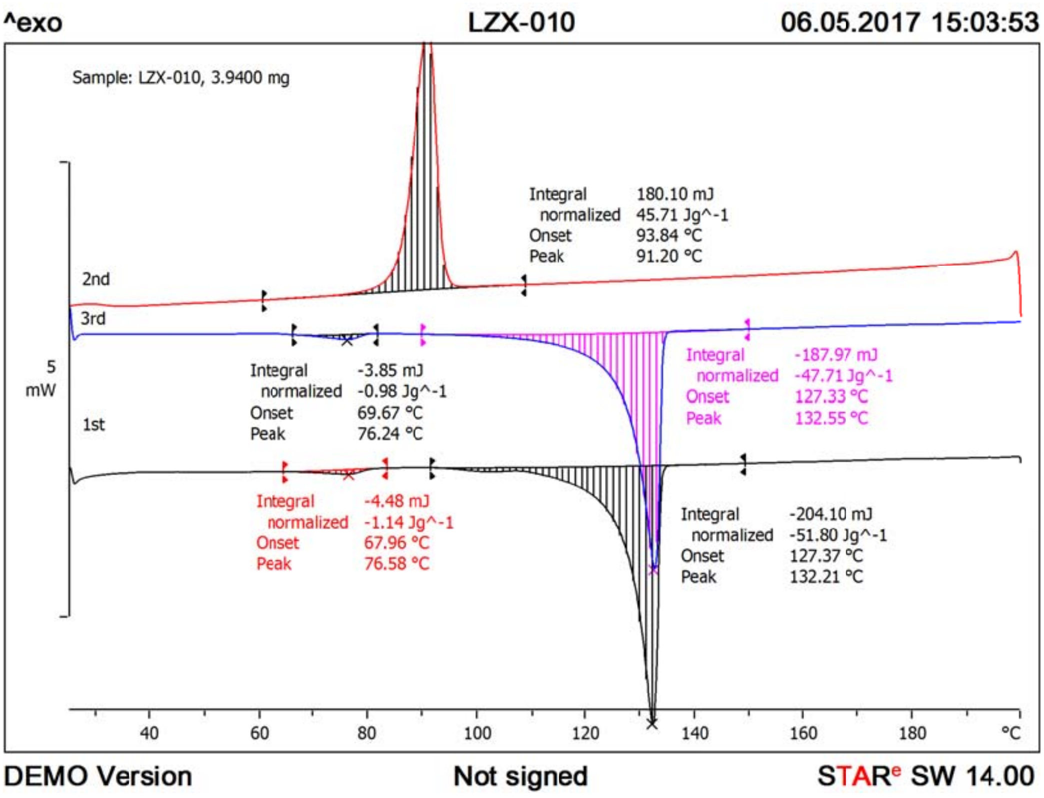

Fig. S5 DSC curve of compound **5b-10**

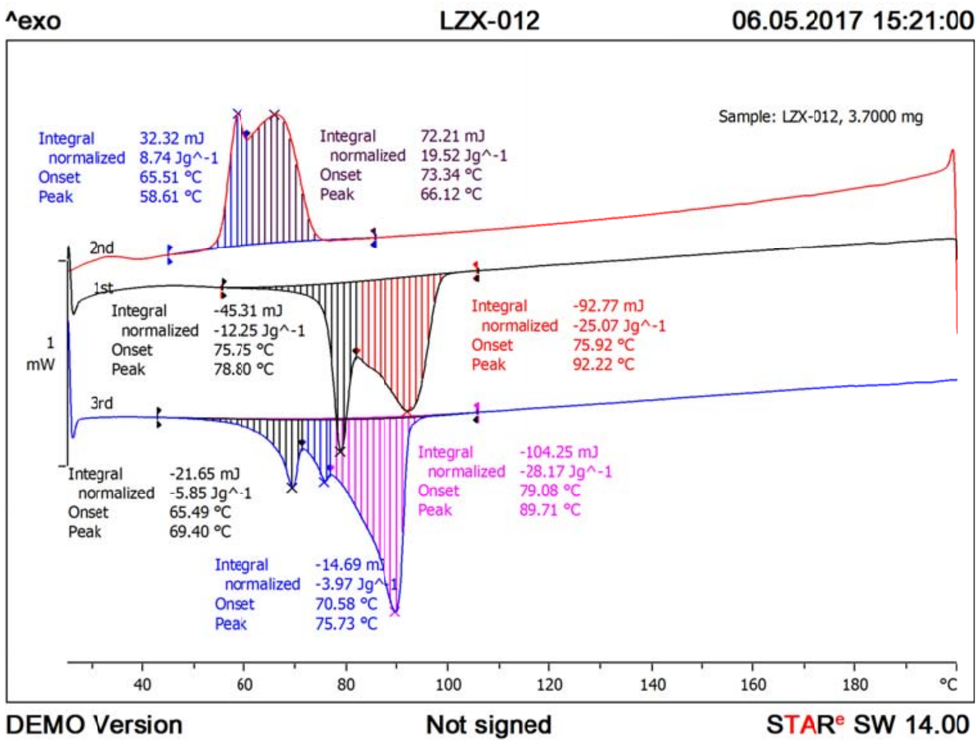

Fig. S6 DSC curve of compound **5c-10**

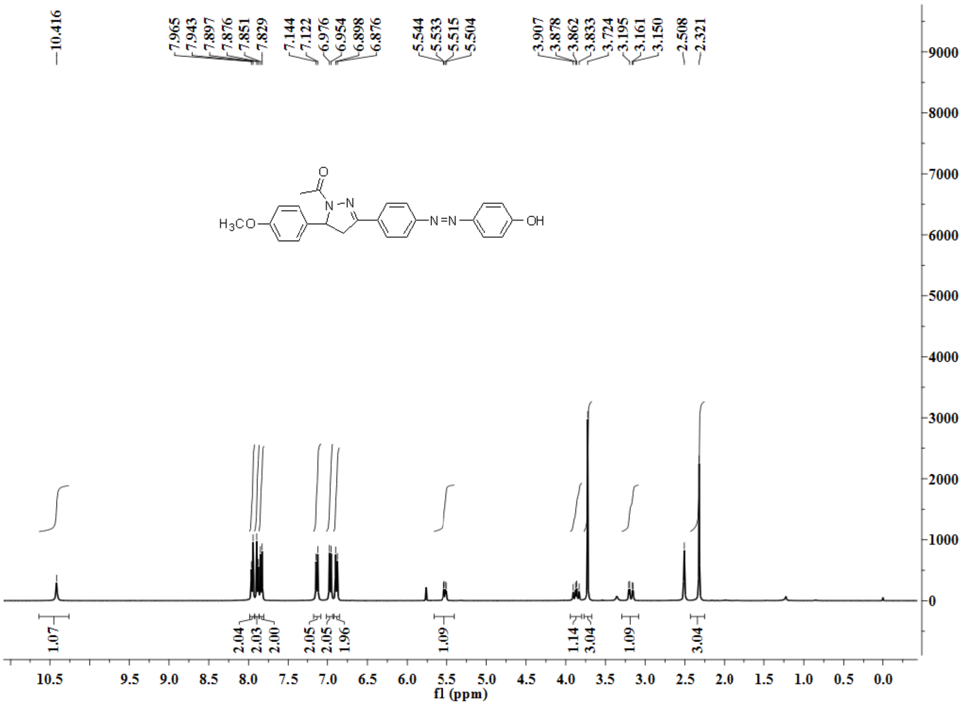

Fig. S7 ^{1}H NMR of compound **2a**

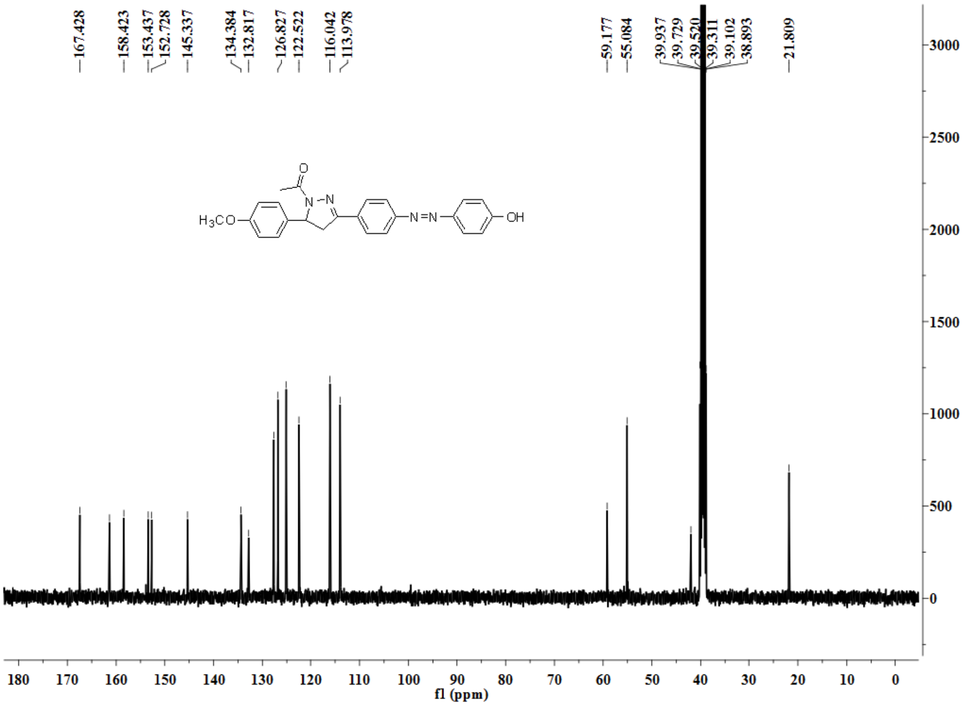

Fig. S8 ^{13}C NMR of compound **2a**

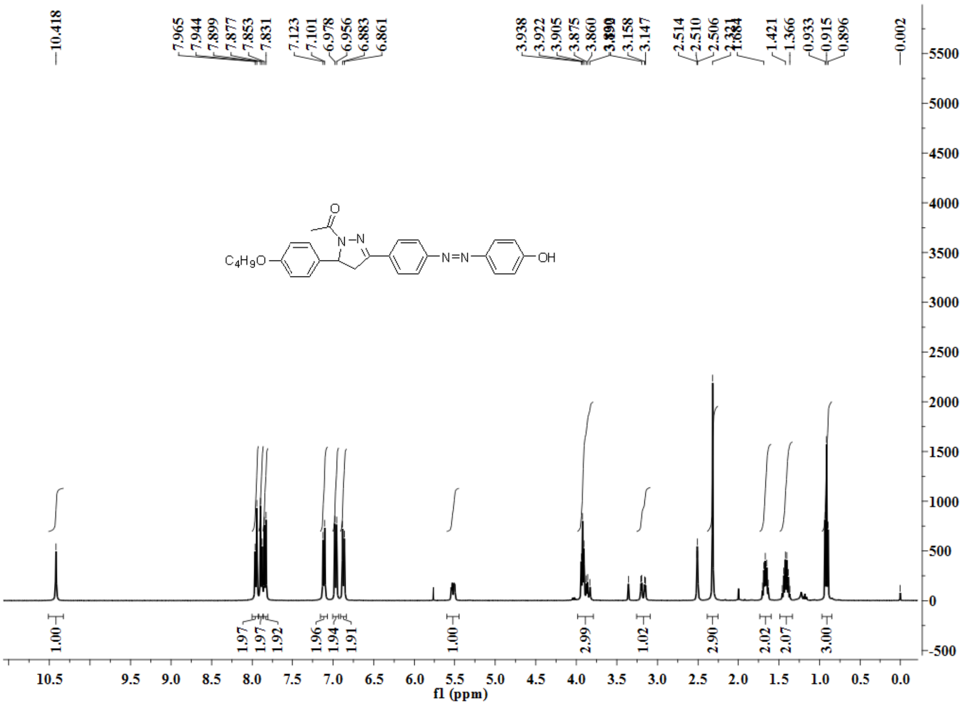

Fig. S9 ^{1}H NMR of compound **2b**

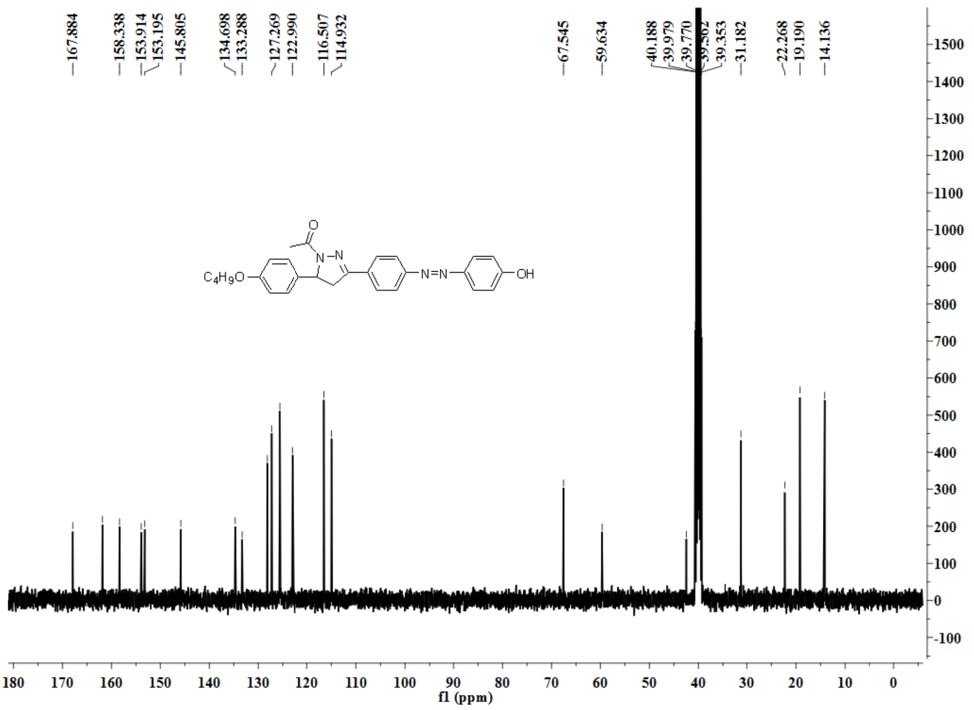

Fig. S10 ^{13}C NMR of compound **2b**

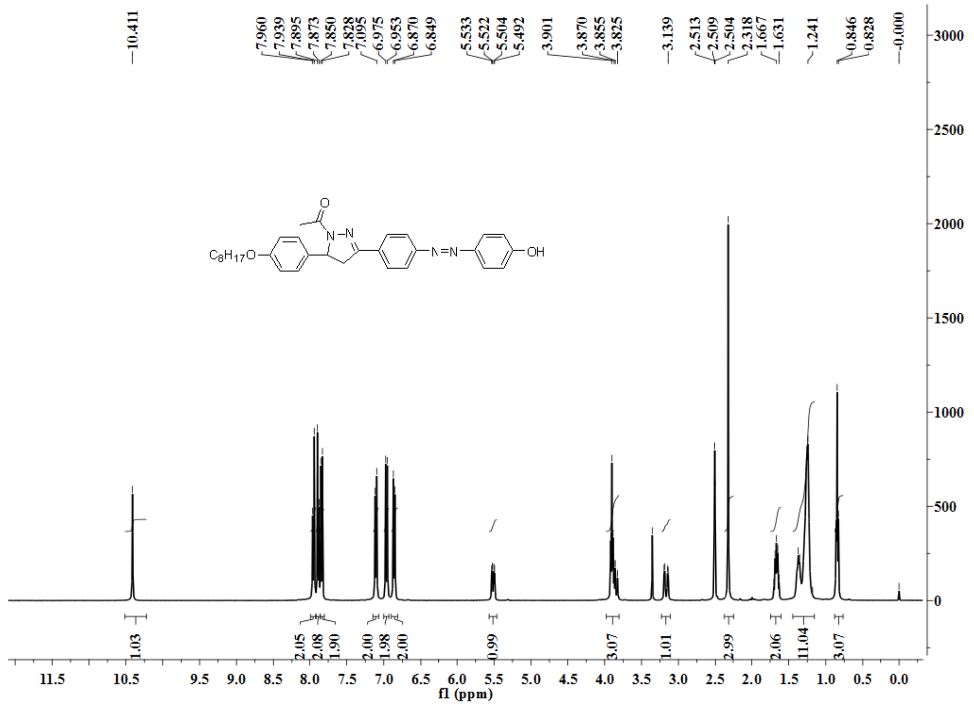

Fig. S11 ^{1}H NMR of compound **2c**

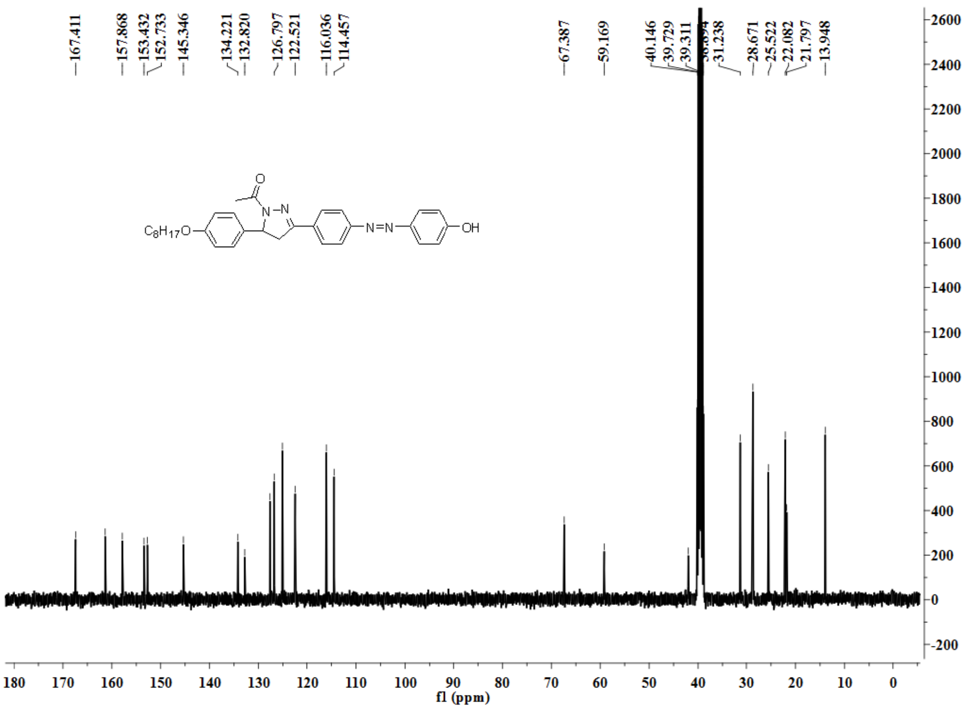

Fig. S12 ^{13}C NMR of compound **2c**

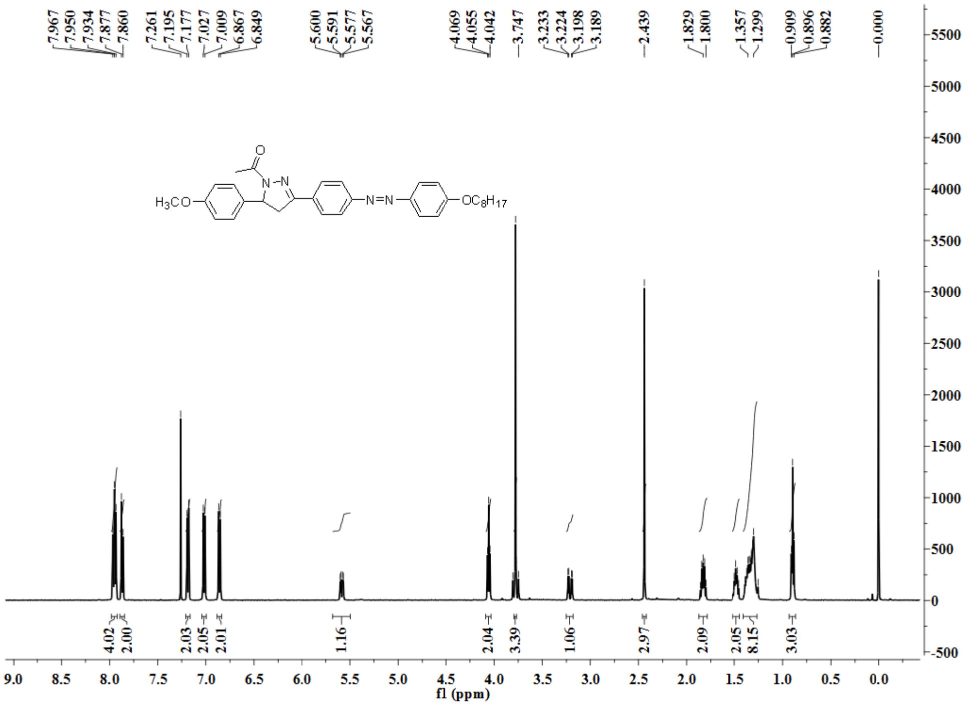

Fig. S13 ^{1}H NMR of compound **3a-8**

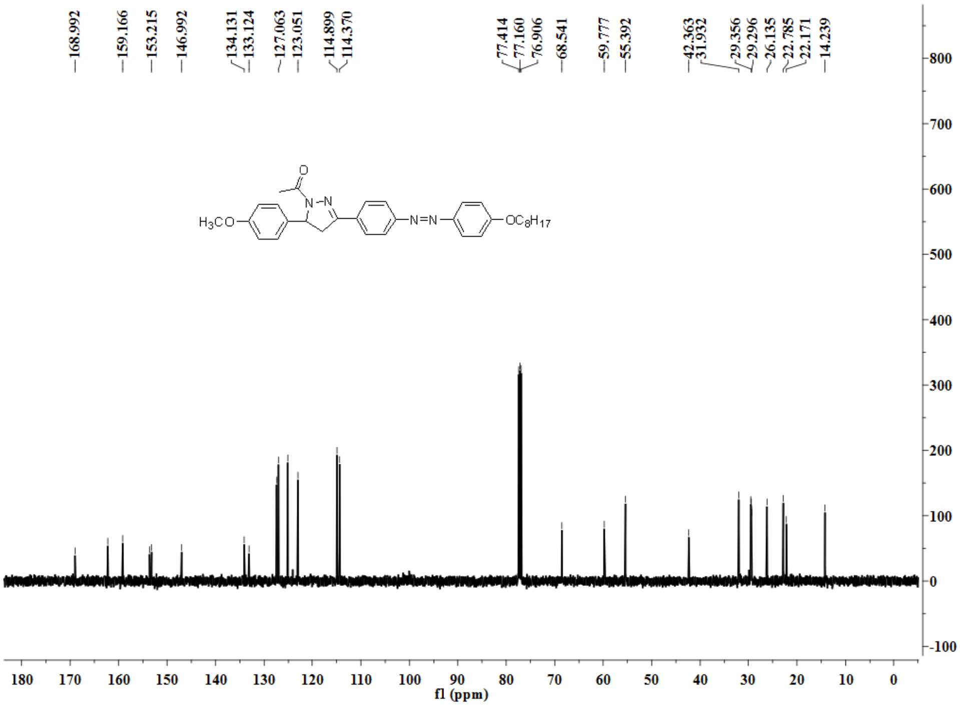

Fig. S14 ^{13}C NMR of compound **3a-8**

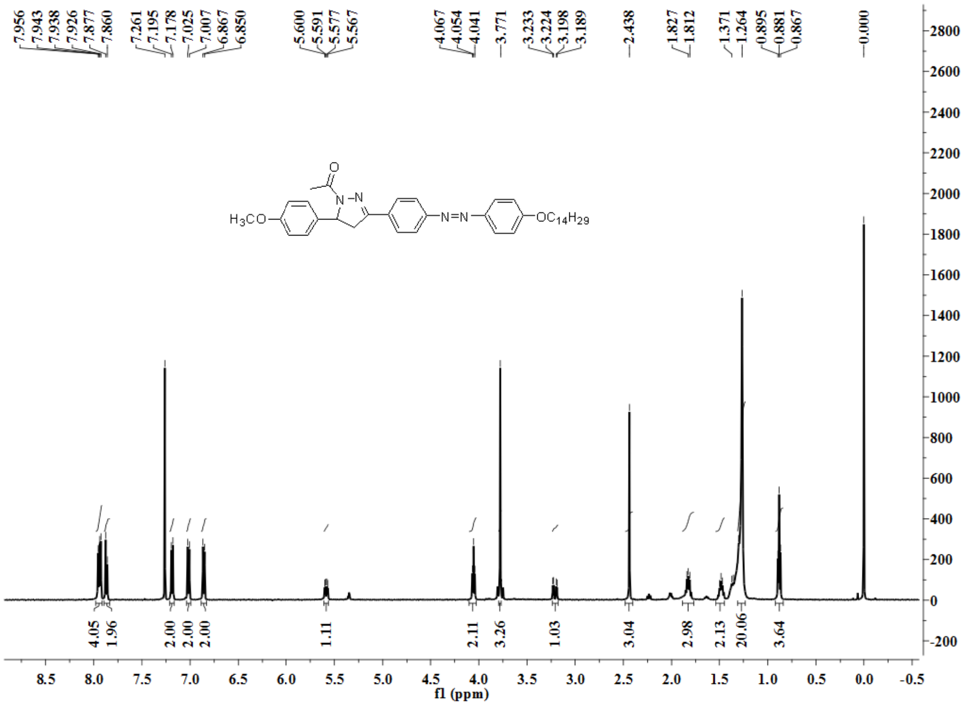

Fig. S15 ^{1}H NMR of compound **3a-14**

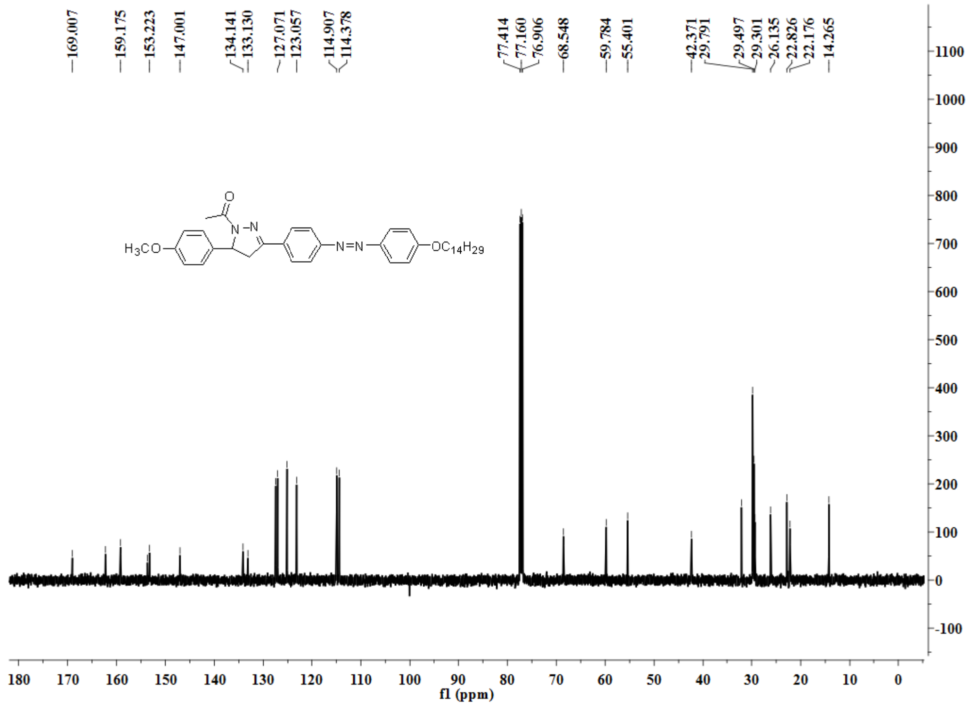

Fig. S16 ^{13}C NMR of compound **3a-14**

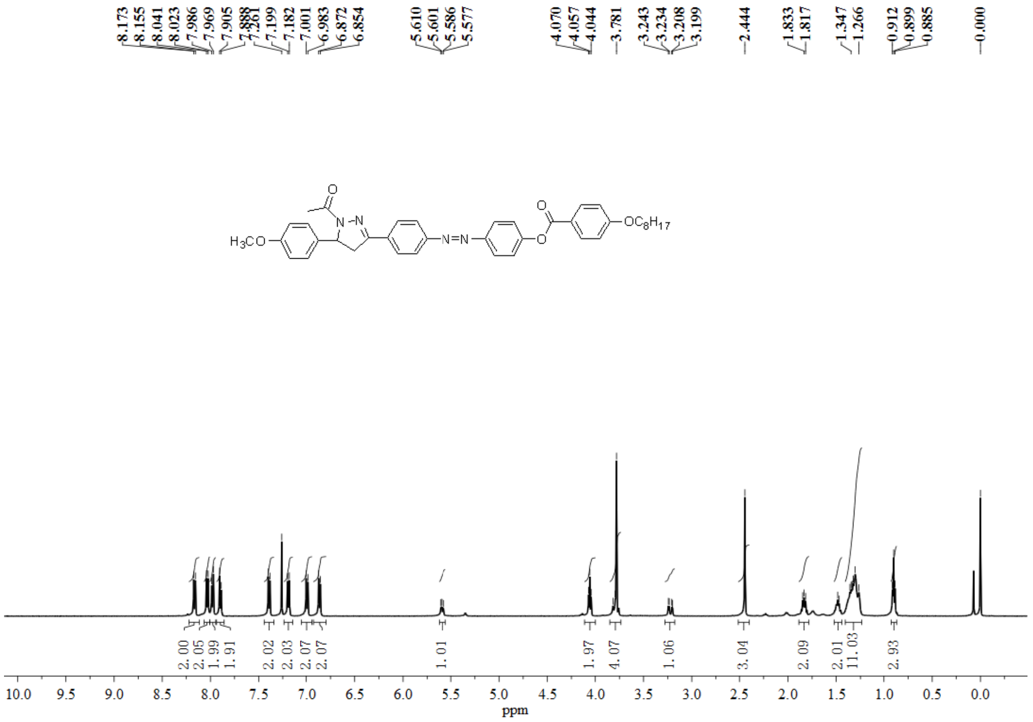

Fig. S17 ^{1}H NMR of compound **5a-8**

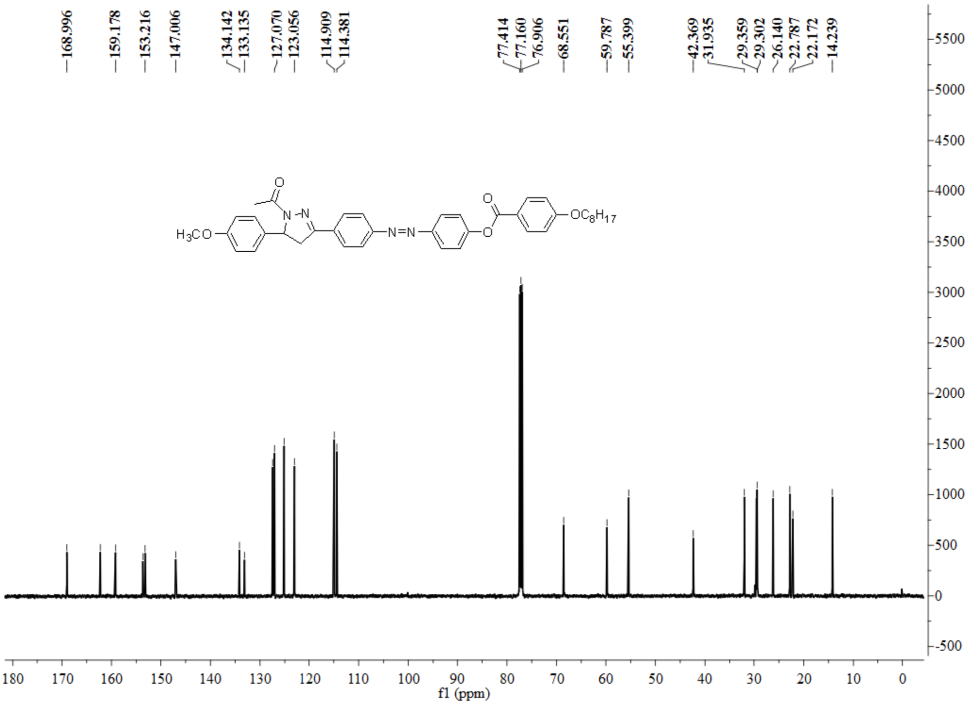

Fig. S18 ^{13}C NMR of compound **5a-8**

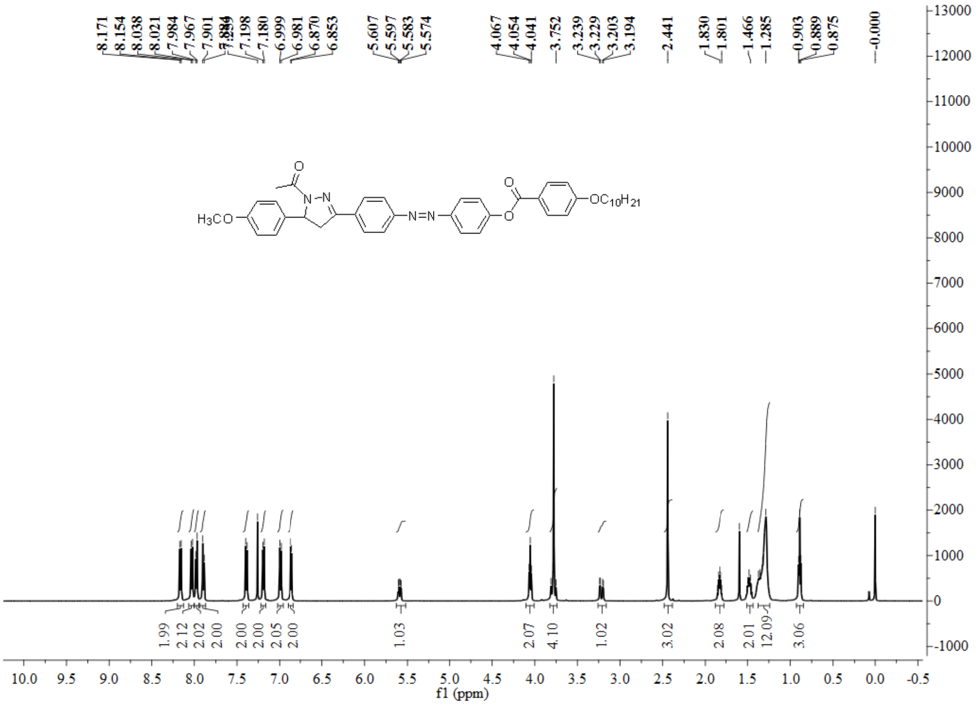

Fig. S19 ^{1}H NMR of compound **5a-10**

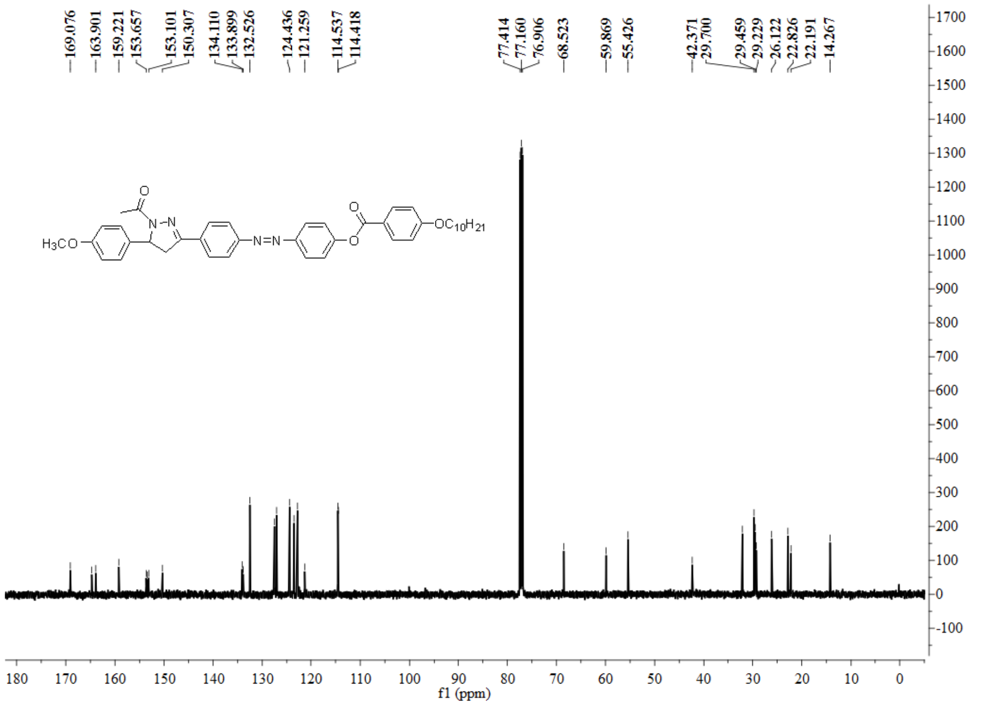

Fig. S20 ^{13}C NMR of compound **5a-10**

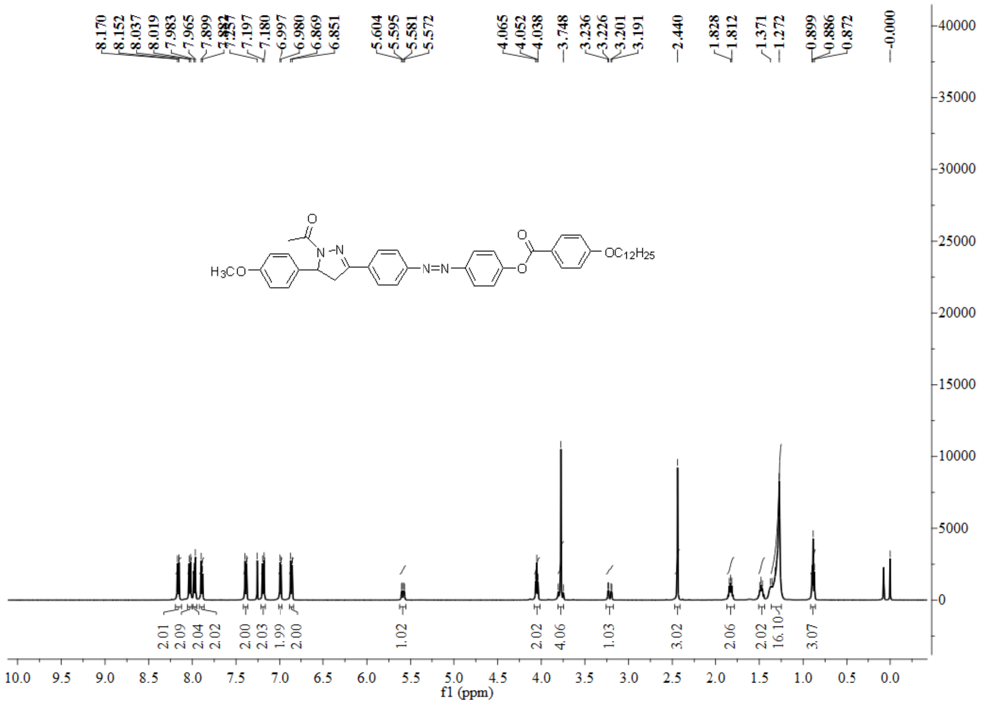

Fig. S21 ^{1}H NMR of compound **5a-12**

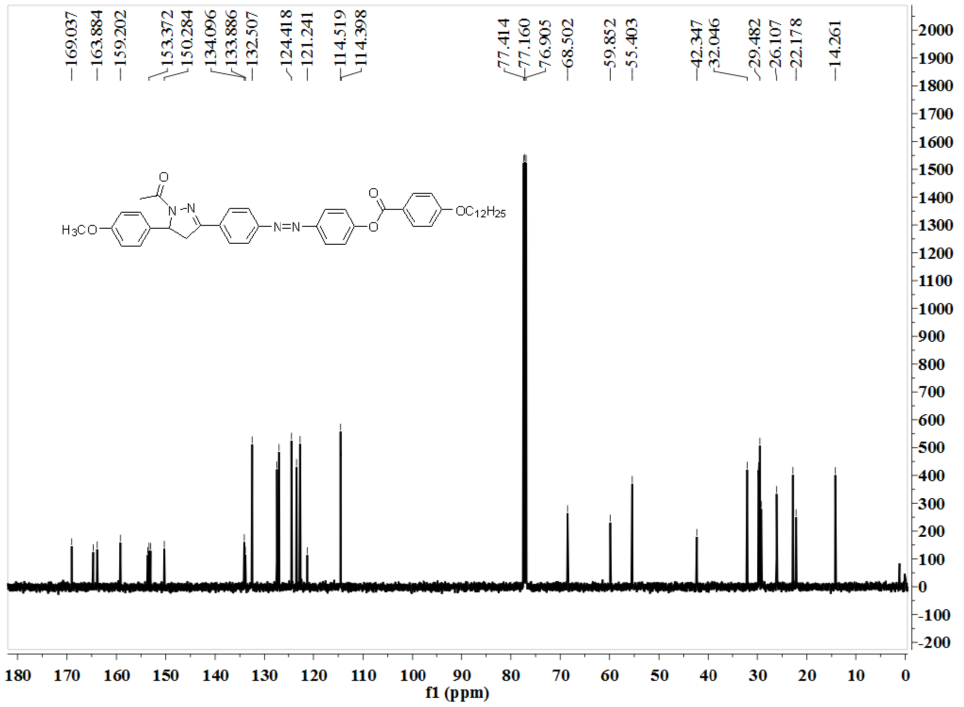

Fig. S22 ^{13}C NMR of compound **5a-12**

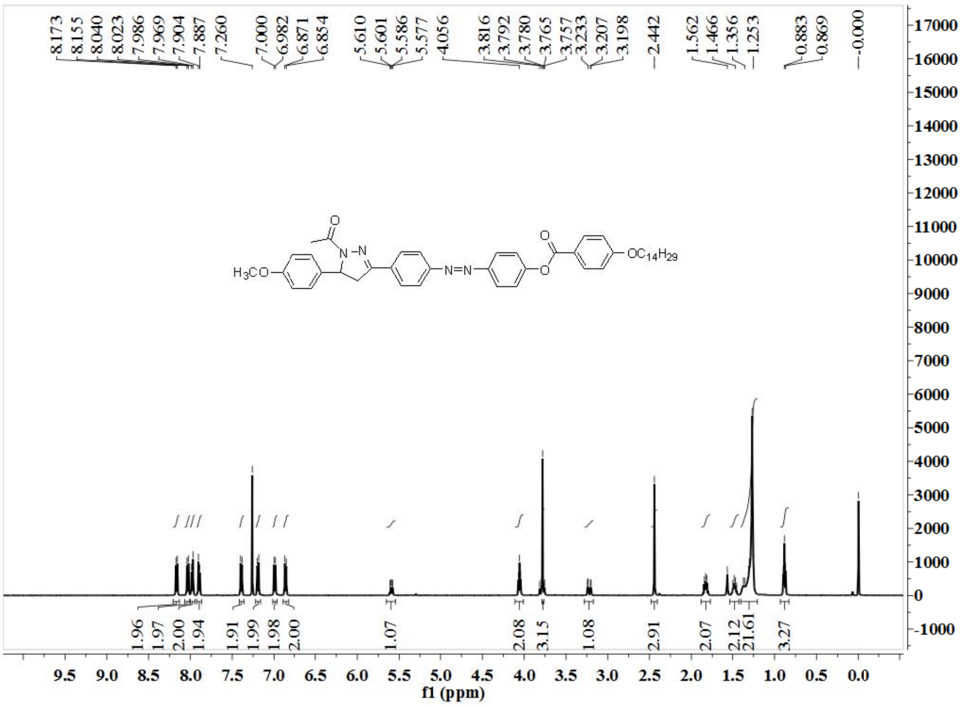

Fig. S23 ^{1}H NMR of compound **5a-14**

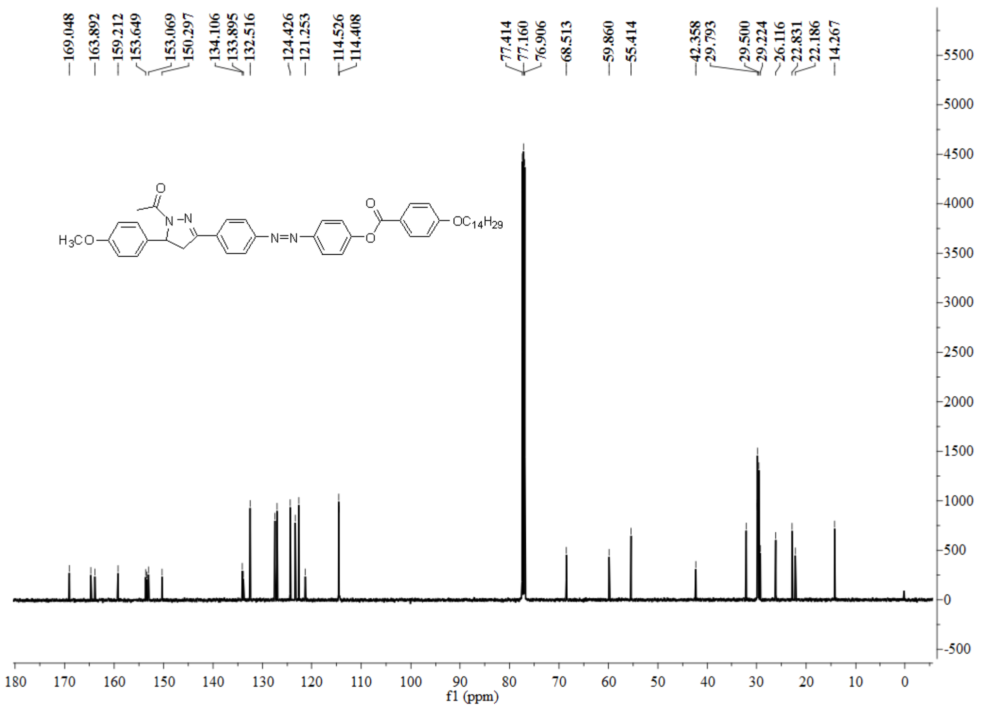

Fig. S24 ^{13}C NMR of compound **5a-14**

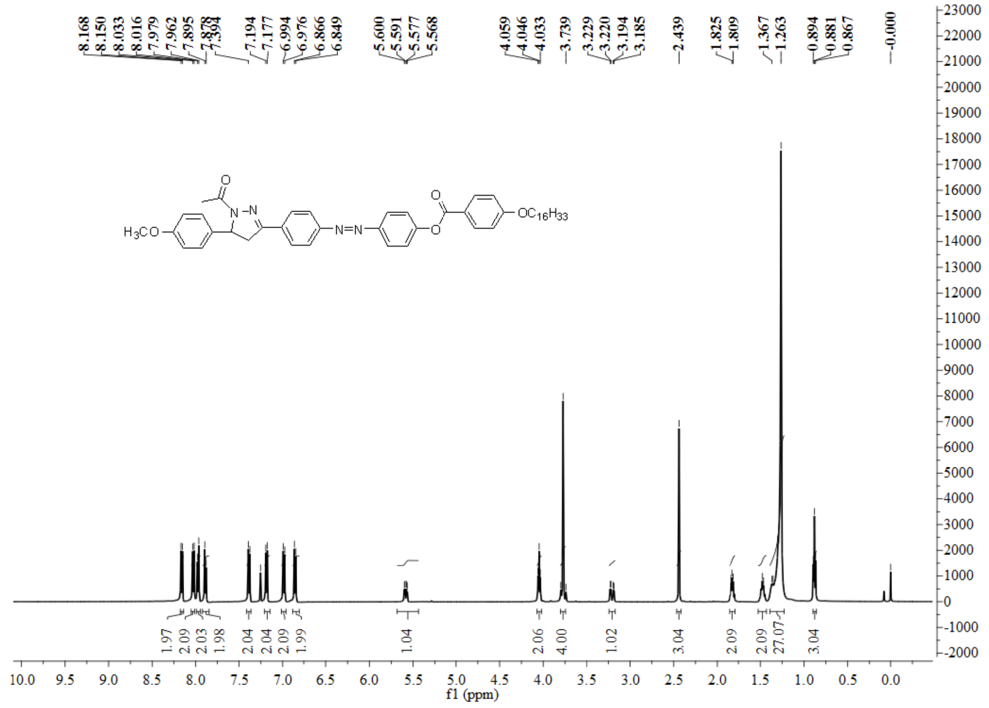

Fig. S25 ^{1}H NMR of compound **5a-16**

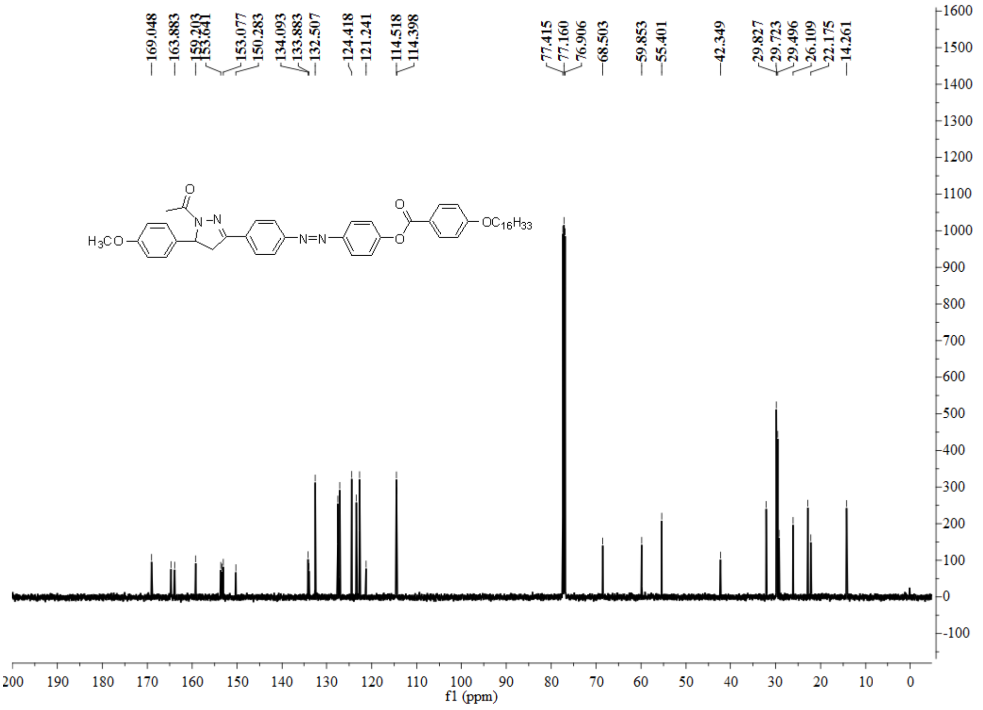

Fig. S26 ^{13}C NMR of compound **5a-16**

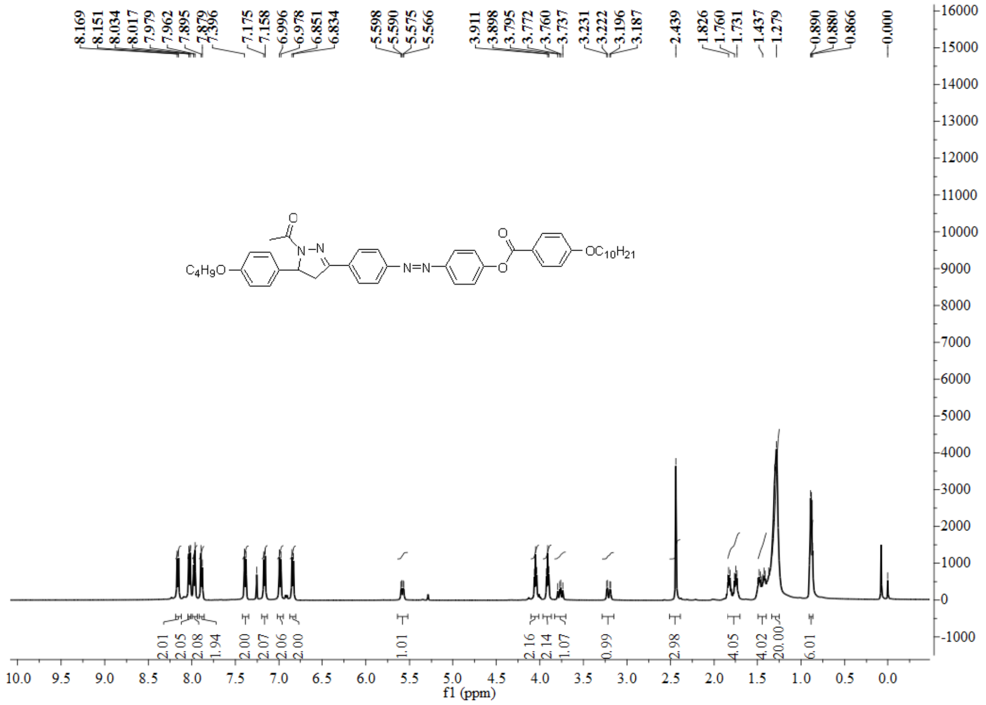

Fig. S27 ^{1}H NMR of compound **5b-10**

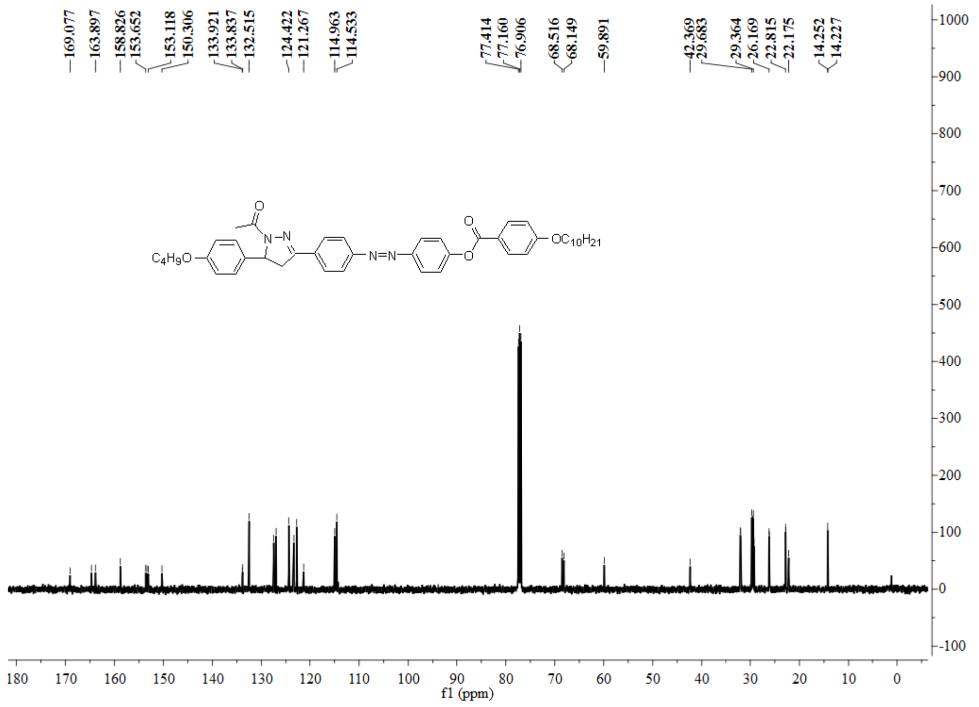

Fig. S28 ^{13}C NMR of compound **5b-10**

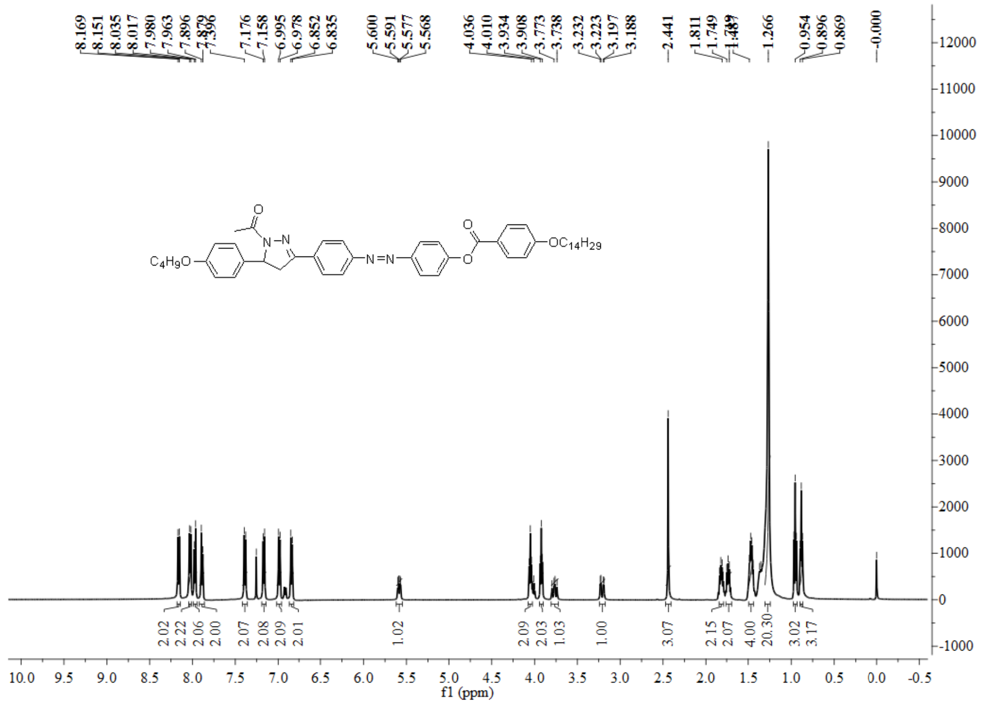

Fig. S29 ^{1}H NMR of compound **5b-14**

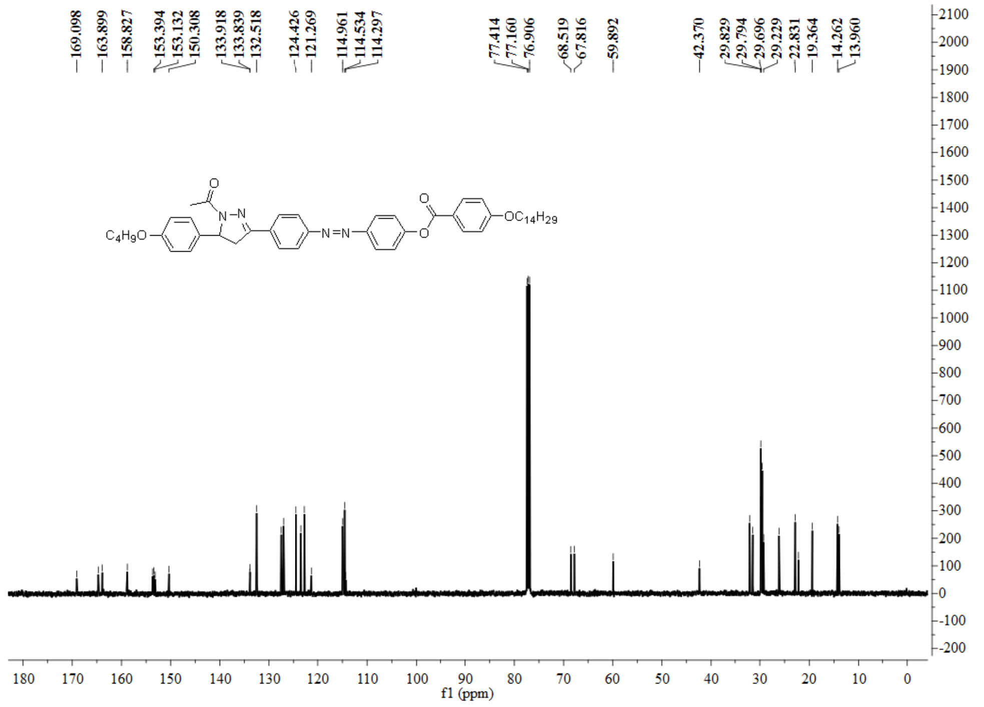

Fig. S30 ^{13}C NMR of compound **5b-14**

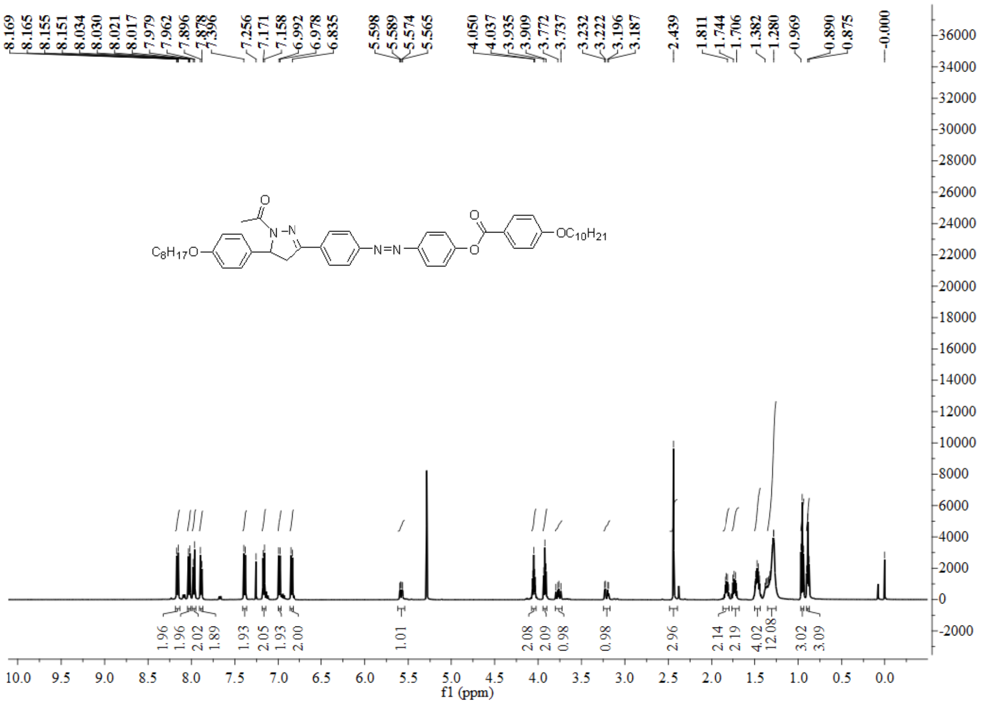

Fig. S31 ^{1}H NMR of compound **5c-10**

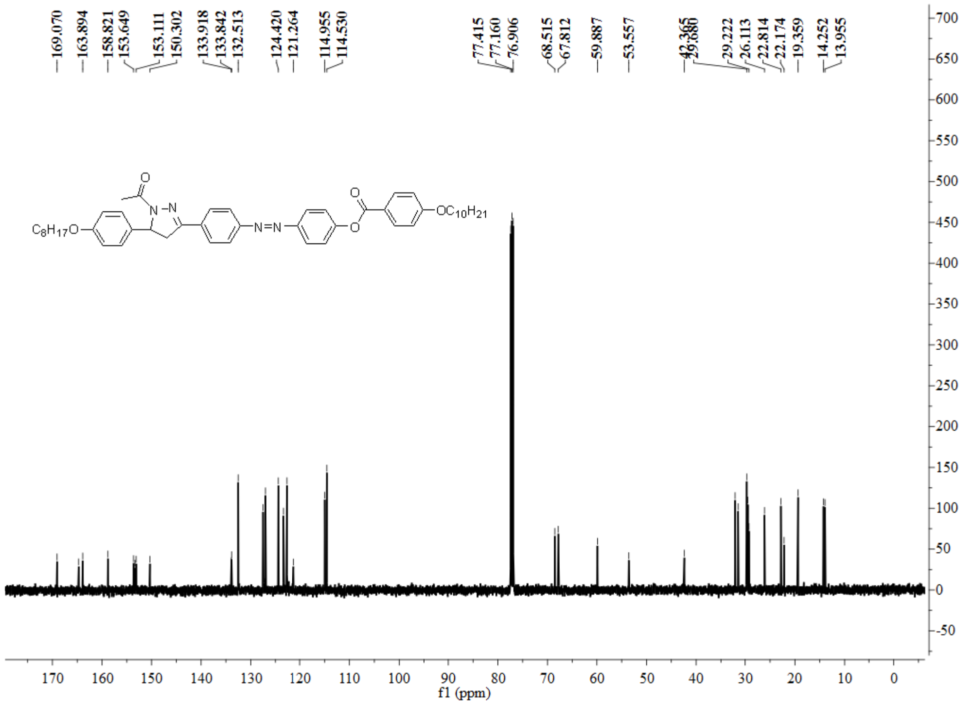

Fig. S32 ^{13}C NMR of compound **5c-10**

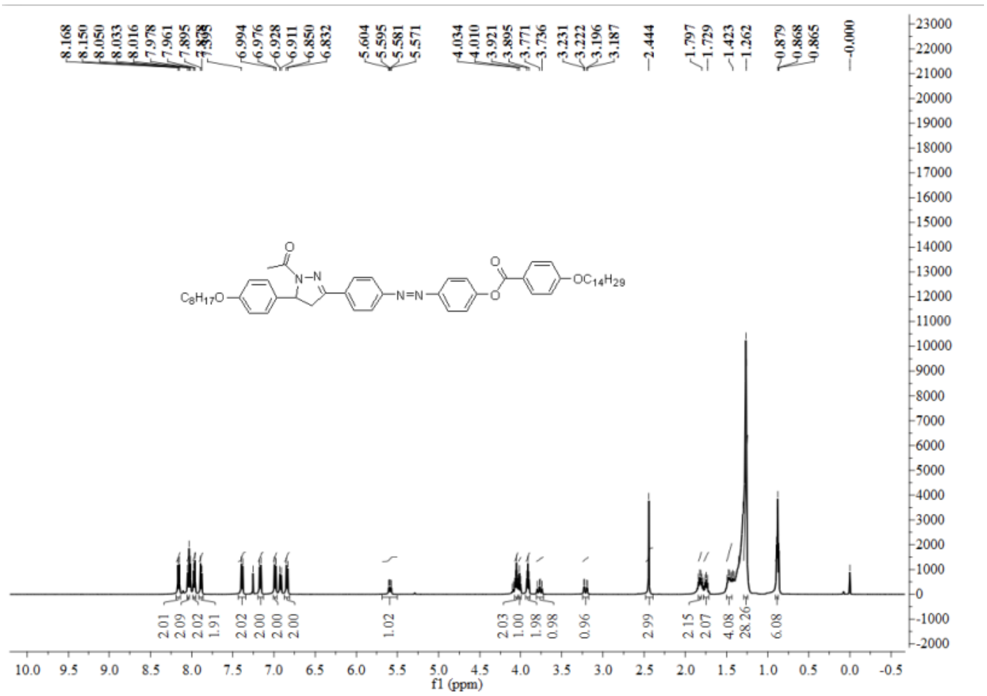

Fig. S33 ^{1}H NMR of compound **5c-14**

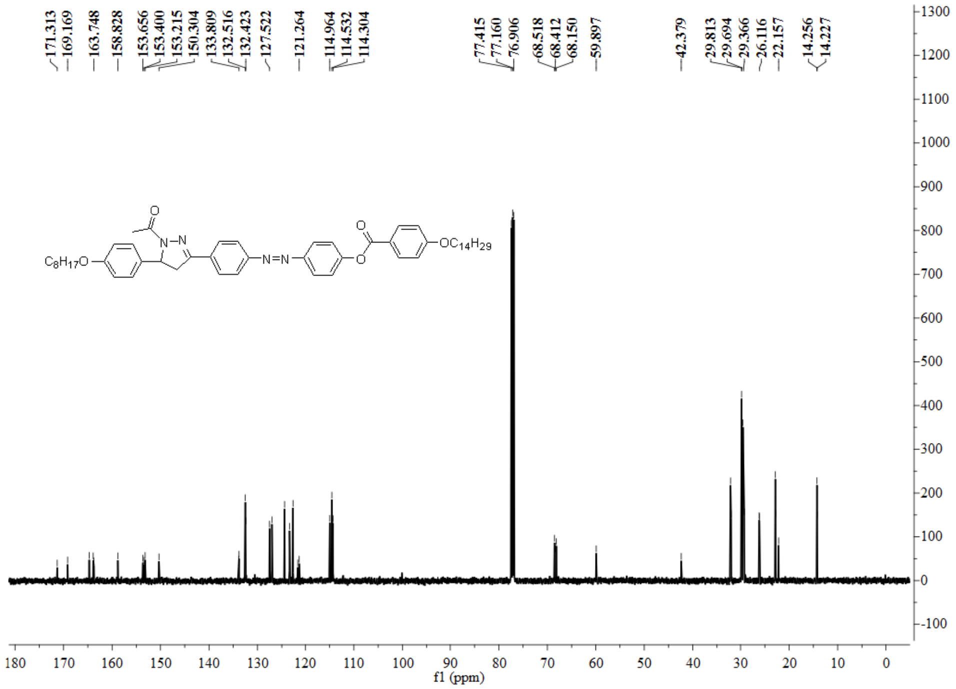

Fig. S34 ^{13}C NMR of compound **5c-14**

Acquisition Parameter

Date of acquisition	2016-09-14T15:33:05.078+08:00
Acquisition method name	D:\Methods\flexControlMethods\gc-RP_100-1500_Da.par
Aquisition operation mode	Reflector
Voltage polarity	POS
Number of shots	500
Name of spectrum used for calibration	
Calibration reference list used	sample

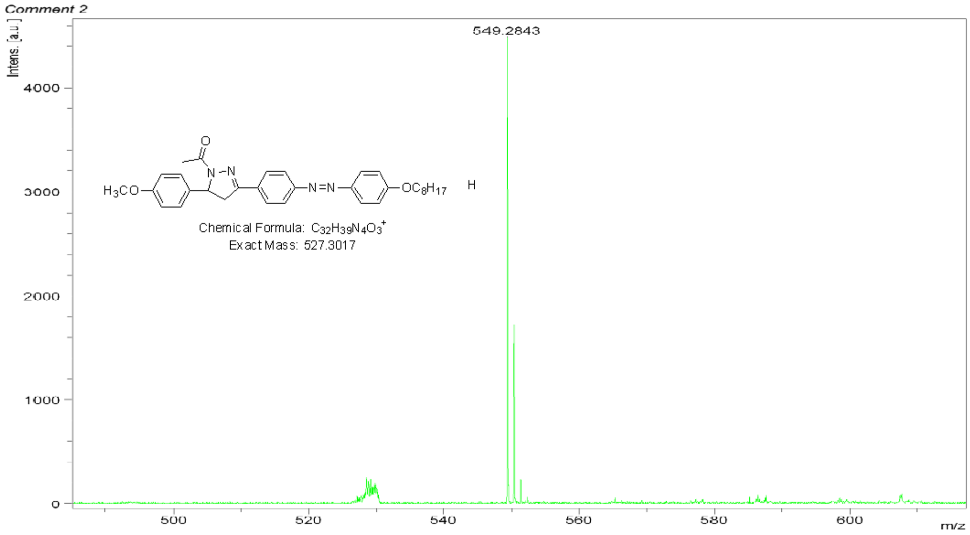

Fig. S35 HRMS of compound **3a-8**

Acquisition Parameter

Date of acquisition	2016-09-14T14:16:26.671+08:00
Acquisition method name	D:\Methods\flexControlMethods\gc-RP_100-1500_Da.par
Aquisition operation mode	Reflector
Voltage polarity	POS
Number of shots	500
Name of spectrum used for calibration	
Calibration reference list used	sample

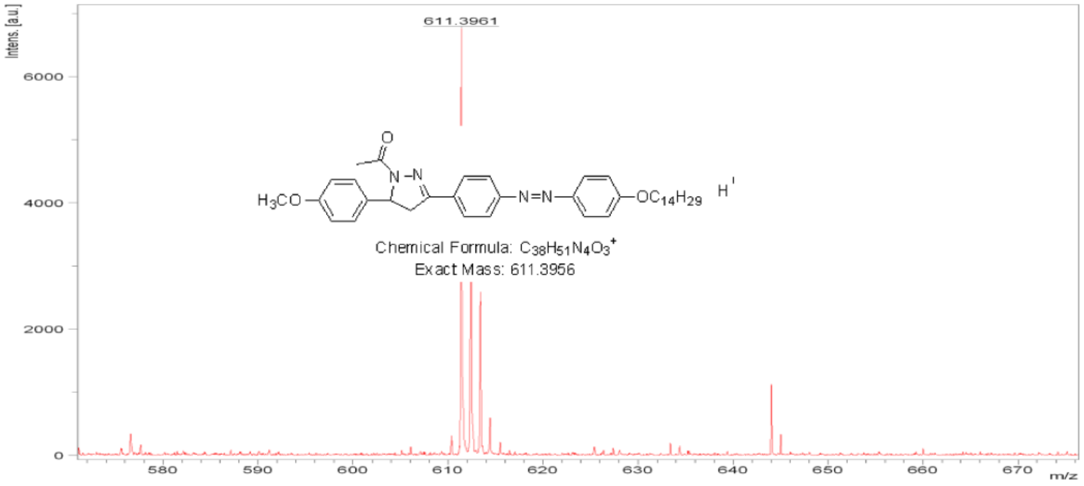

Fig. S36 HRMS of compound **3a-14**

Acquisition Parameter

Date of acquisition	2016-09-14T14:16:26.671+08:00
Acquisition method name	D:\Methods\flexControlMethods\gc-RP_100-1500_Da.par
Aquisition operation mode	Reflector
Voltage polarity	POS
Number of shots	500
Name of spectrum used for calibration	
Calibration reference list used	sample

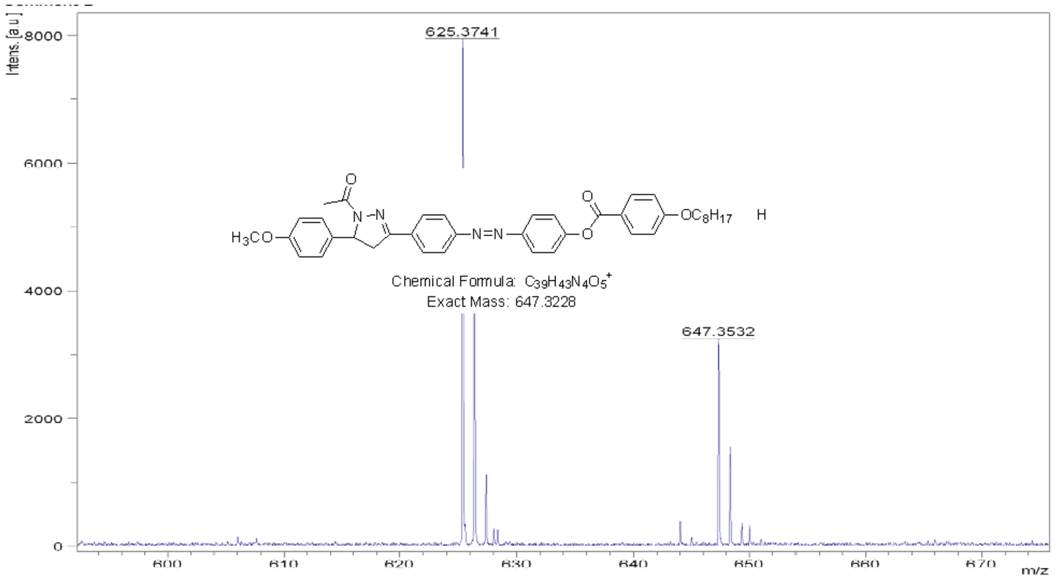

Fig. S37 HRMS of compound **5a-8**

Acquisition Parameter

Date of acquisition	2016-09-14T14:35:01.531+08:00
Acquisition method name	D:\Methods\flexControlMethods\gc-RP_100-1500_Da.par
Aquisition operation mode	Reflector
Voltage polarity	POS
Number of shots	500
Name of spectrum used for calibration	
Calibration reference list used	sample

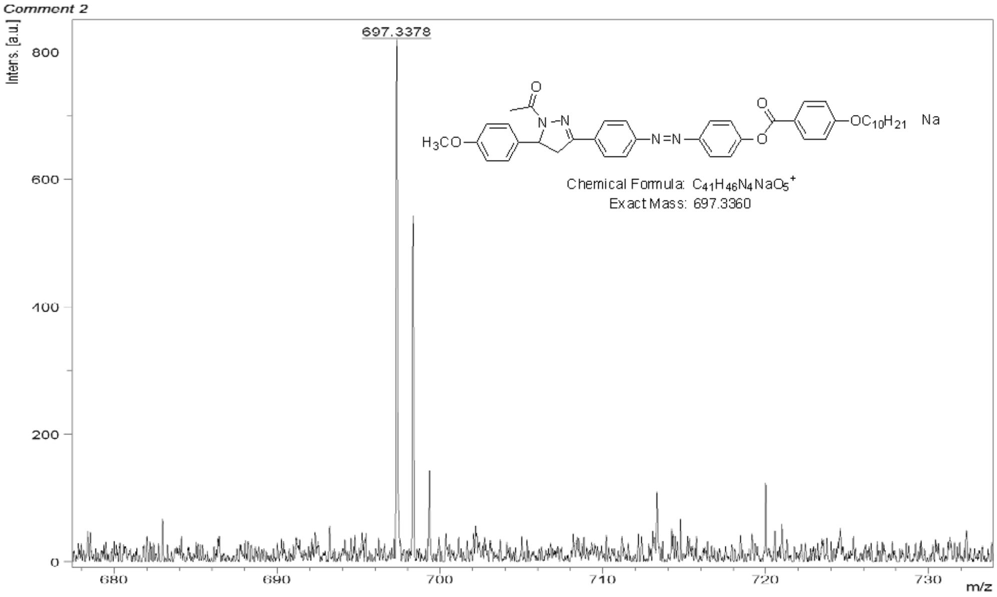

Fig. S38 HRMS of compound **5a-10**

Acquisition Parameter

Date of acquisition	2016-09-14T14:39:14.062+08:00
Acquisition method name	D:\Methods\flexControlMethods\gc-RP_100-1500_Da.par
Aquisition operation mode	Reflector
Voltage polarity	POS
Number of shots	500
Name of spectrum used for calibration	
Calibration reference list used	sample

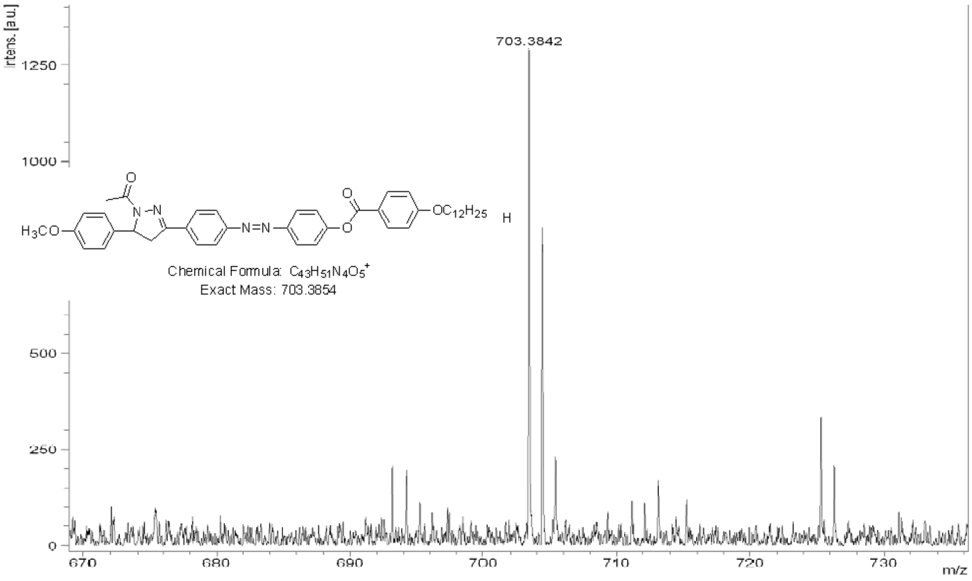

Fig. S39 HRMS of compound **5a-12**

Mass Spectrum SmartFormula Report

Analysis Info

Analysis Name	J:\Àî³¯Ï¼\¸ß·Ö±æ\wangyongqiang-P2.d	Acquisition Date	2017/3/7 11:38:21
Method	tune_low 50-500.m	Operator	NWU
Sample Name	< No Sample >	Instrument / Ser#	micrOTOF-Q II 10280
Comment			

Acquisition Parameter

Source Type	ESI	Ion Polarity	Positive	Set Nebulizer	0.4 Bar
Focus	Not active	Set Capillary	4500 V	Set Dry Heater	180 °C
Scan Begin	50 m/z	Set End Plate Offset	-500 V	Set Dry Gas	4.0 l/min
Scan End	1000 m/z	Set Collision Cell RF	110.0 Vpp	Set Divert Valve	Source

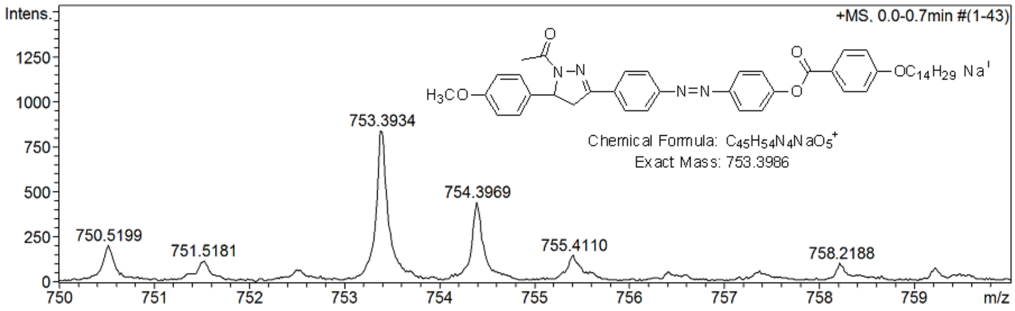

Fig. S40 HRMS of compound **5a-14**

Acquisition Parameter

Date of acquisition	2016-09-14T14:39:58.562+08:00
Acquisition method name	D:\Methods\flexControlMethods\gc-RP_100-1500_Da.par
Aquisition operation mode	Reflector
Voltage polarity	POS
Number of shots	500
Name of spectrum used for calibration	
Calibration reference list used	sample

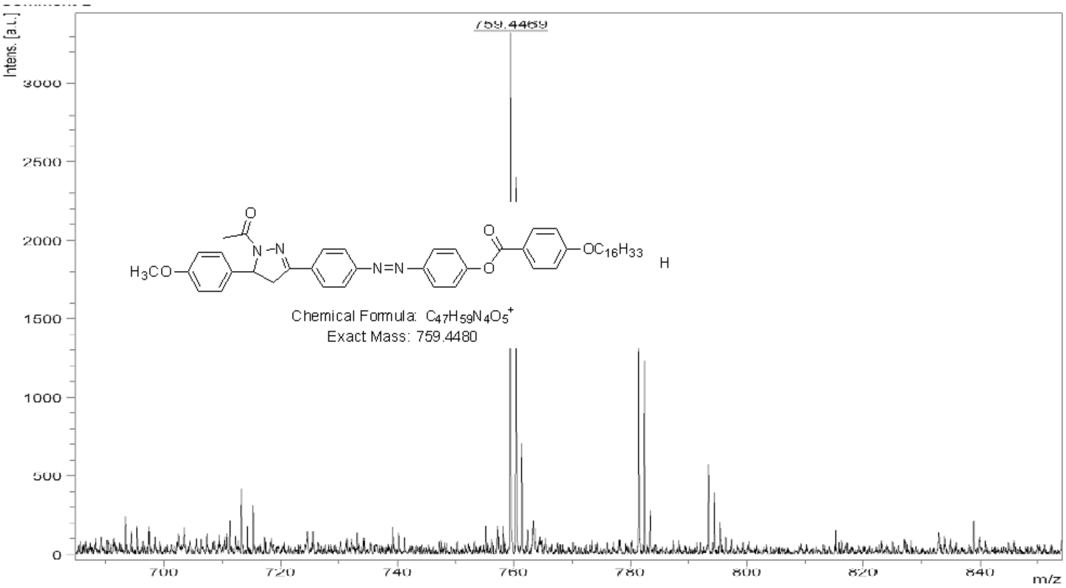

Fig. S41 HRMS of compound **5a-16**

Mass Spectrum SmartFormula Report

Analysis Info

Analysis Name	J:\ÀÎ³¯Ï¼\¸ß·Ö±æ\wangyongqiang-P3.d	Acquisition Date	2017/3/7 11:40:32
Method	tune_low 50-500.m	Operator	NWU
Sample Name	< No Sample >	Instrument / Ser#	micrOTOF-Q II 10280
Comment			

Acquisition Parameter

Source Type	ESI	Ion Polarity	Positive	Set Nebulizer	0.4 Bar
Focus	Not active	Set Capillary	4500 V	Set Dry Heater	180 °C
Scan Begin	50 m/z	Set End Plate Offset	-500 V	Set Dry Gas	4.0 l/min
Scan End	1000 m/z	Set Collision Cell RF	110.0 Vpp	Set Divert Valve	Source

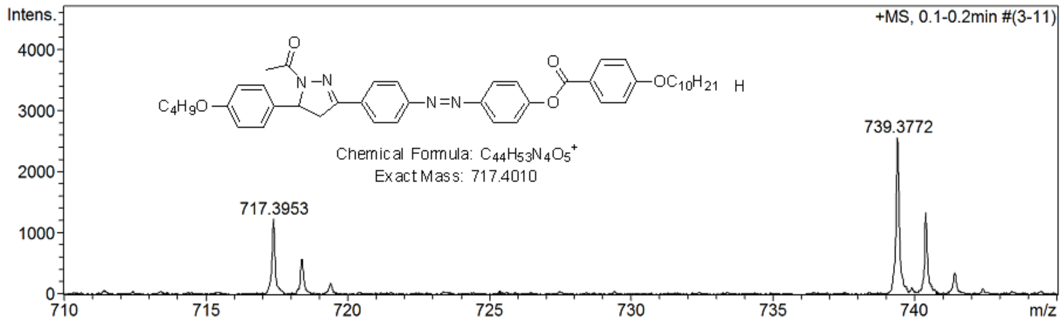

Fig. S42 HRMS of compound **5b-10**

Mass Spectrum SmartFormula Report

Analysis Info

Analysis Name	J:\ÀÎ³¯Ï¼\¸ß·Ö±æ\wangyongqiang-P4.d	Acquisition Date	2017/3/7 11:41:29
Method	tune_low 50-500.m	Operator	NWU
Sample Name	< No Sample >	Instrument / Ser#	micrOTOF-Q II 10280
Comment			

Acquisition Parameter

Source Type	ESI	Ion Polarity	Positive	Set Nebulizer	0.4 Bar
Focus	Not active	Set Capillary	4500 V	Set Dry Heater	180 °C
Scan Begin	50 m/z	Set End Plate Offset	-500 V	Set Dry Gas	4.0 l/min
Scan End	1000 m/z	Set Collision Cell RF	110.0 Vpp	Set Divert Valve	Source

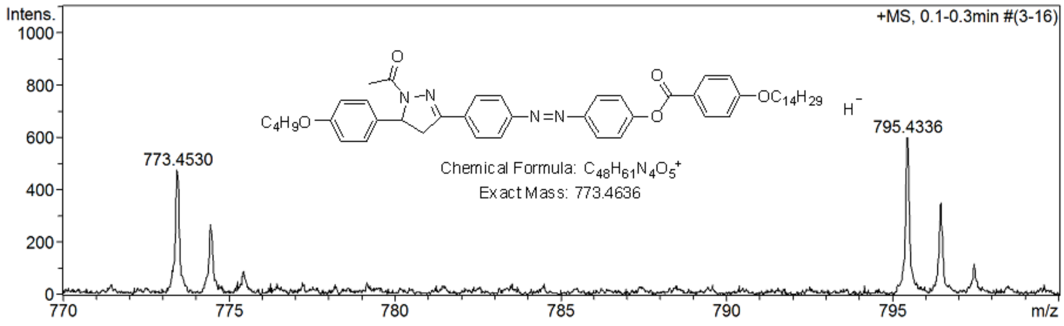

Fig. S43 HRMS of compound **5b-14**

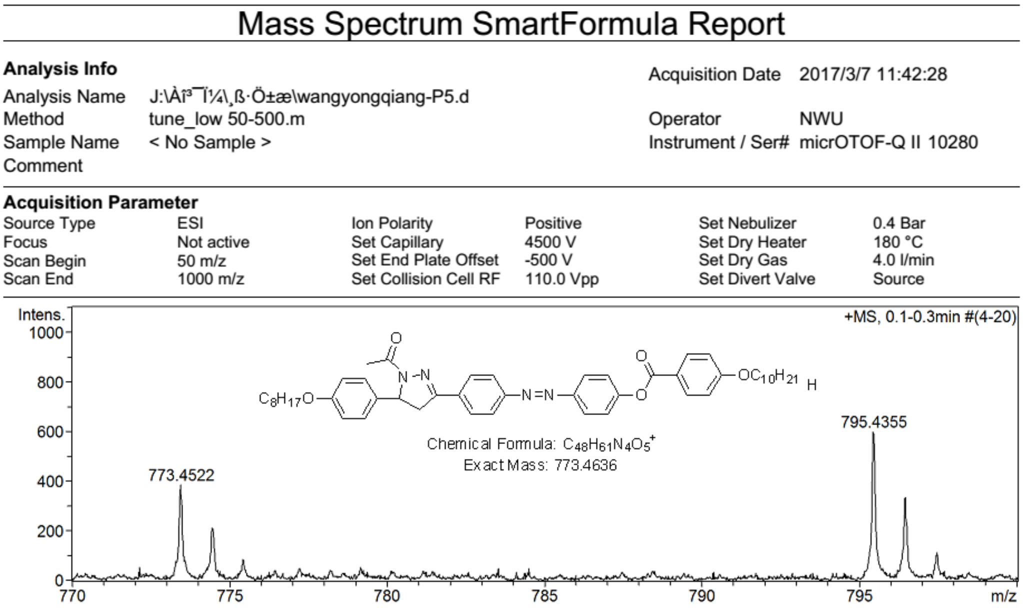

Fig. S44 HRMS of compound **5c-10**

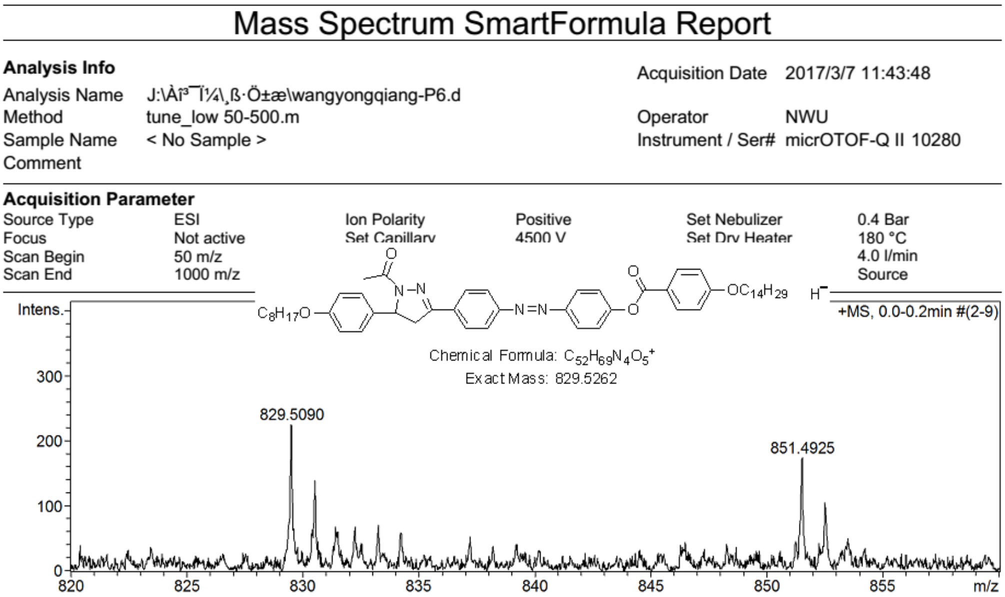

Fig. S45 HRMS of compound **5c-14**
